# Transgenerational Epigenetic Inheritance Under Environmental Stress by Genome-Wide DNA Methylation Profiling in Cyanobacterium

**DOI:** 10.3389/fmicb.2018.01479

**Published:** 2018-07-04

**Authors:** Lang Hu, Peng Xiao, Yongguang Jiang, Mingjie Dong, Zixi Chen, Hui Li, Zhangli Hu, Anping Lei, Jiangxin Wang

**Affiliations:** ^1^Shenzhen Key Laboratory of Marine Bioresource and Eco-environmental Science, Shenzhen Engineering Laboratory for Marine Algal Biotechnology, Guangdong Provincial Key Laboratory for Plant Epigenetics, College of Life Sciences and Oceanography, Shenzhen University, Shenzhen, China; ^2^Nanshan District Key Lab for Biopolymers and Safety Evaluation, College of Materials Science and Engineering, Shenzhen University, Shenzhen, China; ^3^Laboratory of Synthetic Microbiology, School of Chemical Engineering and Technology, Tianjin University, Tianjin, China

**Keywords:** cyanobacteria, adaptation, environmental stress, DNA methylation, transgenerational epigenetic inheritance

## Abstract

Epigenetic modifications such as DNA methylation are well known as connected with many important biological processes. Rapid accumulating evidence shows environmental stress can generate particular defense epigenetic changes across generations in eukaryotes. This transgenerational epigenetic inheritance in animals and plants has gained interest over the last years. Cyanobacteria play very crucial role in the earth, and as the primary producer they can adapt to nearly all diverse environments. However, few knowledge about the genome wide epigenetic information such as methylome information in cyanobacteria, especially under any environment stress, was reported so far. In this study we profiled the genome-wide cytosine methylation from a model cyanobacterium *Synechocystis* sp. PCC 6803, and explored the possibility of transgenerational epigenetic process in this ancient single-celled prokaryote by comparing the DNA methylomes among normal nitrogen medium cultivation, nitrogen starvation for 72 h and nitrogen recovery for about 12 generations. Our results shows that DNA methylation patterns in nitrogen starvation and nitrogen recovery are much more similar with each other, significantly different from that of the normal nitrogen. This study reveals the difference in global DNA methylation pattern of cyanobacteria between normal and nutrient stress conditions and reports the evidence of transgenerational epigenetic process in cyanobacteria. The results of this study may contribute to a better understanding of epigenetic regulation in prokaryotic adaptation to and survive in the ever changing environment.

## Introduction

Epigenetic modifications, heritable chemical additions to DNA or histones which are associated with gene expression but do not alter the primary DNA sequence, are well known as connected with many important processes (Law and Jacobsen, 2010). DNA methylation is one of the most important epigenetic modifications and catalyzed by DNA-specific methyltransferase which transfers methyl group from general substrate, *S*-adenosylmethionine, to specific DNA sequences. This epigenetic modification is usually related to regulation of gene expression, genomic imprinting, X chromosome inactivation, chromosome stability and so on in eukaryotes (Feng et al., 2010).

DNA methylation mainly refers to 5-methylcytosine (thereafter m^5^C) in eukaryotes. In mammals most m^5^C exist within CG context, and methyltransferases DNMT3 and DNMT1 are responsible for establishment and maintenance of the DNA methylation, respectively. In higher plants, m^5^C could exist in CG, CHG, and CHH context. Establishment of the methylation is conducted by methyltransferase DRM2, and two methyltransferases, MET1 and CMT3, are involved in maintenance of the methylation in higher plants (Chan et al., 2005; Law and Jacobsen, 2010). Bisulfite can convert cytosine into uracil but cannot alter m^5^C (Patterson et al., 2011). The whole genome bisulfite sequencing (thereafter WGBS) technology has been widely used in exploring the global pattern of m^5^C in eukaryotes (i.e., Lister et al., 2009; Carvalho et al., 2012). In addition, m^4^C can also show partially resistance to the bisulfite-mediated deamination, therefore WGBS indeed could be used to identify both m^5^C and m^4^C (Vilkaitis and Klimasauskas, 1999).

Besides methylation by DNA methyltransferases, demethylation could also influence the global DNA methylation pattern in mammals and plants. Deaminases (Aid/Apobec), glycosylase (Mbd4), thymine DNA glycosylase (Tdg), and unidentified apyrimidinic lyase are needed for active demethylation in mammals, while bifunctional 5-methylcytosine glycosylases (DME/ROS1) are needed for active demethylation in higher plants (Ikeda and Kinoshita, 2009).

In bacteria, besides m^5^C, N6-methyladenine (m^6^A) and N4-methylcytosine (m^4^C) also exist. Unlike in mammals and plants, DNA methylation in bacteria is believed to be part of the restriction/modification system, which prevents bacteria from being self-digested (Wilson and Murray, 1991). Taking *Escherichia coli* genome as an example, three DNA methyltransferase genes exist, one of which is clustered with its cognate restriction enzyme gene and the other two encode solitary methyltransferases. In bacteria, DNA methyltransferases may also have other physiological significances. The methyltransferase, Dam, involves in distinguishing newly synthesized DNA from the old DNA (Palmer and Marinus, 1994) and takes part in DNA repair (Pukkila et al., 1983). In *Caulobacter crescentus*, the methyltransferase CcrM acts as a regulator for cell cycle (Reisenauer et al., 1999). Single molecule real-time (SMRT) sequencing technology could be used to directly detect m^6^A and m^4^C with high degree of accuracy and sensitivity (Flusberg et al., 2010). With this technology, many bacterial and archaeal species were investigated for their global m^6^A and m^4^C distribution patterns (Clark et al., 2012; Fang et al., 2012; Murray et al., 2012; Lluch-Senar et al., 2013; Blow et al., 2016). Besides SMRT sequencing, WGBS has also been applied into bacterial such as *E. coli* to determine the global m^5^C distribution (Kahramanoglou et al., 2012).

As an ancient group of photosynthetic bacteria, cyanobacteria plays very crucial role in the earth as the primary producer in the ecosystem. They could be found in many diverse ecological habitats, and survive under adverse environments such as deserts, polar region, and hot springs (Whitton and Potts, 2000; Stomp et al., 2007). Some cyanobacteria species may form water bloom, threatening the health of human (Catherine et al., 2013). Based on sequence homology, some methyltransferase encoding genes have been identified in cyanobacteria. For example, there are nine genes possibly encoding DNA methyltransferase in *Anabaena* sp. PCC 7120 (Matveyev et al., 2001). Type II restriction/modification system and solitary methyltransferase genes were also predicted in *N. punctiforme* genome (Meeks et al., 2001). In *Synechocystis* sp. PCC 6803 (thereafter *Synechocystis*), a model cyanobacterium, three genes encoding solitary methyltransferases, *mbpA*, *sll0729*, and *synMI*, exist (Kaneko et al., 1995, 1996; Scharnagl et al., 1998). Some limited DNA methylation studies have also been conducted in cyanobacteria. Restriction analysis and quantitative estimation of methylated bases in filamentous and unicellular cyanobacterial DNA shows the presence of methyladenine in the GATC sequence (Padhy et al., 1988). Enzyme digestion experiments in two *Anabaena* species indicates that DNA from both species is methylated, but that no gross change in methylation occurs during heterocyst formation (Adams, 1988). Recently, global DNA methylation pattern study has been conducted in *Synechocystis* and more methyltransferases were identified (Hagemann et al., 2018).

Many studies have reported external environment stress could influence the epigenetic processes in eukaryotes (Gao et al., 2010; Lira-Medeiros et al., 2010; Kou et al., 2011; Menzel et al., 2011; Navarro-Martin et al., 2011; Yu et al., 2013). Furthermore, rapid accumulating evidence shows that environmental stress could generate particular defense epigenetic changes across generations in eukaryotes (Hauser et al., 2011; Heard and Martienssen, 2014; Skinner, 2014). This transgenerational epigenetic inheritance has gained interest over the last years and addressed the importance of transgenerational inheritance for adaptation to ever changing environment and for practical applications. For instance, in *Oryza sativa* L., it has been reported chronic nitrogen deficiency could induce global change in DNA methylation in leaf-tissue of the stressed plant, and part of the change could be stably inherited to its non-stress suffered progenies and improve the resistance to nitrogen deficiency in progenies (Kou et al., 2011). In contrast, in bacteria few studies concerned about the influence of external environment on global DNA methylation pattern, much less the defense transgenerational epigenetic inheritance.

While nutrients are important to microbes, seasonal depletion and repletion of nutrients in the environment are common in nature. In some habitats, nitrogen, one of the most important essential nutrients, is seriously scarce for cyanobacteria to survive (Elser et al., 1990; Vitousek and Howarth, 1991; Conley et al., 2009; Thomas et al., 2015). However, cyanobacteria have evolved sophisticated mechanisms to cope with the nitrogen deficiency. For example, most cyanobacteria could utilize various alternative forms of nitrogen source under nitrogen deficiency (Flores and Herrero, 2005). Moreover, some cyanobacteria could biologically fix nitrogen from air (Wolk, 1996). Considering that DNA methyltransferase is widespread in this group (Kaneko et al., 1995, 1996; Scharnagl et al., 1998; Matveyev et al., 2001; Meeks et al., 2001), it is possible for cyanobacteria to deal with the stress through defense alteration in DNA methylation as in plant (Kou et al., 2011). However, knowledge about the whole-genome DNA methylation in cyanobacteria is still poor, and defense epigenetic modifications such as DNA methylation in this group, especially under any environment stress, remain a mystery.

In this study, DNA from a model single-celled model cyanobacterium *Synechocystis* cultured under normal nitrogen (sample as NC), nitrogen starved for 72 h (N72), and nitrogen recovered (NR) which was derived from N72 but thereafter NR for 12 days was bisulfite converted for next generation sequencing, and the DNA methylation patterns were compared among the three samples. Questions proposed to answer are: (i) whether widely spread mC exists in *Synechocystis*? (ii) whether the DNA methylation could be influenced by nutrient depletion or not? and (iii) if influenced whether the altered pattern is inheritable and how? Results indicate that nitrogen deficiency stress can induce DNA methylation alternations, and gives the evidence of transgenerational epigenetic process, inheritance of DNA methylation pattern from N72 to NR, in cyanobacteria. Our current data will contribute to a better understanding of the biological function, epigenetic adaptation to ever changing environment, and even the evolution of DNA methylation in prokaryotes.

## Materials and Methods

### Strains and Culture Conditions

*Synechocystis* sp. PCC 6803 (thereafter *Synechocystis*) was provided kindly by Professor Weiwen Zhang in Tianjin University, China. All cells were cultured autotrophically at a constant photon flux density of 30 μmol photons m^-2^ s^-1^ on a rotary shaker at 30°C. The BG11 medium which contains 1.5 g L^-1^ NaNO_3_ (Rippka et al., 1979), N^0^ BG11 medium (modified BG11 containing no NaNO_3_), and N^1/3^ BG11 medium (modified BG11 containing only 0.5 g L^-1^ NaNO_3_) were used.

### Nitrogen Starvation and Nitrogen Recovery

*Synechocystis* cells were cultured in BG11 medium, and when OD_730_ reached to 0.8 cells were collected as normal nitrogen sample (NC). Some cells taken from NC were transferred into N^0^ BG11 medium with OD_730_ = 0.8 and starved for 72 h, then cells were collected as nitrogen starved sample (N72). Some cells derived from N72 were transferred back into BG11 medium and cultured for 6 days with initial OD_730_ = 0.05. After 6 days some cells were collected and retransferred into BG11 medium with initial OD_730_ = 0.05 for another cultivation of 6 days. Finally, cells were collected as nitrogen recovery sample (NR). Cells for whole genome bisulfite sequencing were all stored in -80°C before processing.

### Extraction of Genomic DNA

Genomic DNA was extracted as described previously (Hu et al., 2015). Briefly, cells were digested with proteinase K (Thermo Fisher Scientific, United States). Lysate was then extracted with mix of phenol (Sinopharm, China) and chloroform (Sinopharm, China). RNA was removed with RNase A (Thermo Fisher Scientific, United States) and DNA was precipitated with isopropanol (Sinopharm, China).

### Extraction of RNA and qRT-PCR

Cells were grinded in liquid nitrogen and then extracted with Trizol (Thermo Fisher Scientific, United States). Genomic DNA was removed by gDNA eraser (Takara, Japan). cDNAs were synthesized using SuperScript VILO^TM^ Master Mix Kit (Thermo Fisher Scientific, United States). The qRT-PCR was conducted on QuantStudio^TM^ 6 flex (Thermo Fisher Scientific, United States). A total of 10 μL qRT-PCR reaction solution was set, containing 5 μL SYBR Premix Ex Taq (Tli RNaseH Plus) (Takara, Japan), 0.2 μL ROX reference dye (Takara, Japan), 200 nM primers, and 10 ng cDNA. The qRT-PCR procedure was set as follows: initial denaturation step of 95°C for 20 s, 40 cycles of 95°C for 10 s, 60°C for 20 s, followed by a melt curve stage. Relative expression of the target genes was determined using 2^-ΔΔ*CT*^ method (Livak and Schmittgen, 2001). The gene *rnpB* was used as internal reference. Means and SDs were calculated from three biological replicates. Primers used were listed in **Supplementary Table S1**.

### Growth in Limited Nitrogen

Cells were cultured in N^1/3^ BG11 with initial OD_730_ = 0.05. OD_730_ was measured every 2 days. Three biological replicates were set. The specific growth rate was calculated as follows:

$$\mu =\frac{\text{In }{OD}_{2}-\text{In }{OD}_{1}}{T_{2}-T_{1}}$$

In the formula, μ represents the specific growth rate for the first 18 days. T_2_ and T_1_ represent day 18 and day 0, and the OD_2_ and OD_1_ represent OD_730_ at day 18 and day 0, respectively. Significant difference in specific growth rate was tested by using *t*-test with the software IBM SPSS Statistics 20.0 (SPSS Inc., Chicago, IL, United States).

### Whole Genome Bisulfite Sequencing

Whole genome bisulfite sequencing was conducted by Novogene Company (China). Briefly, sample DNA spiked with λ phage genome DNA was fragmented into 200–300 bp with ultra-sonication (Covaris S220 System, Thermo Fisher Scientific, United States). Barcodes were ligated to the DNA fragments, and then bisulfite converted with EZ DNA Methylation Gold Kit (Zymo Research, United States). The bisulfite conversion was conducted according to the protocol provided by the kit. λ phage genome DNA was included to help assess the methylation rate (bisulfite conversion rate). The constructed library was quantified with Qubit 2.0 (Thermo Fisher Scientific, United States), and diluted to 1 ng μL^-1^. The length of the inserted fragment of the library was checked with Agilent 2100 Bioanalyzer system (Agilent). In order to ensure the quality of the library, the effective concentration of the library is kept to ≥2 nM. Sequencing of clustering of the index-coded DNA fragments was conducted on Hiseq sequencer (Illumina, United States) with PE125 sequencing strategy.

### Quality Control of Sequencing

Reads were first pre-processed through in-house perl scripts, and the software Trimmomatic was used for the raw data trimming. The Trimmomatic processing parameters were set as follows: SLIDING WINDOW was set to 4:15. Both LEADING and TRAILING were set to 3. ILLUMINACLIP:adapter.fa:2:30:7:1:true was applied. Clean reads were retrieved as follows: Firstly, reads were scanned for adapter sequence and those reads with adapter sequences were filtered out. Then percentage of undefined bases (N bases) in each read was calculated, and the reads with undefined base percentage higher than 5% were also removed. Lastly, those reads with low quality (PHRED score ≤ 20, and percentage of the low quality bases ≥ 50%) were removed. The remaining reads were considered as clean reads, and all subsequent analyses were based on clean reads.

Details such as data size, read length, sequencing depth, median coverage and so on can be found in results section.

### Reads Mapping to the Reference Genome and Identification of Sites for Methylcytosine

Bismark software (Krueger and Andrews, 2011) was used to perform the alignment of reads to the reference genome (NCBI Bioproject ID: PRJNA80481). To identify the sites for methylcytosine, we treated the sum of methylated reads as a binomial random variable with methylation rate (bisulfite conversion rate), and the fdr *q*-value of each mC candidate was calculated with R script. Only sites with ≥5× coverage and fdr *q*-value ≤ 0.05 were used in analyses.

To calculate methylation level of mC sites, we first calculated the uncorrected methylation level for each cytosine site, and then corrected it with bisulfite non-conversion rate. The uncorrected methylation level for each cytosine site was calculated as follows:

$${ML}_{\left(\text{uncorrected}\right)}=\frac{{reads}_{\left(mC\right)}}{{reads}_{\left(mC\right)}+{reads}_{\left(C\right)}}$$

In the formula, ML_*(uncorrected)*_ represents the uncorrected methylation level for the given site. The reads_*(mC)*_ and reads_*(C)*_ represent the reads with mC and reads with mC or cytosine in the given site, respectively.

Then uncorrected methylation level was further corrected with the bisulfite non-conversion rate and the corrected methylation level for each cytosine site was calculated as follows:

$$ML=\frac{{ML}_{\left(\text{uncorrected}\right)}-R}{1-R}$$

In the formula, ML represents the corrected methylation level and R represents the bisulfite non-conversion rate.

### Differentially Methylated Regions Analysis

Differentially methylated regions (DMRs) were identified using sliding-window approach with the software package swDMR^1^. The window length was set to 200 bp, and the step length was set to 40 bp. Fisher test was conducted to interrogate DMRs. Those genes located in DMRs are defined as differentially methylated genes.

### KEGG Enrichment Analysis of DMR-Related Genes

KEGG is a database resource for understanding high-level functions and utilities of the biological system (Kanehisa et al., 2008). KOBAS software package was used to test the statistical enrichment of DMR-related genes in KEGG pathways (Wu et al., 2006).

### Comparison of Methylome Data With the Published Transcriptomic Results

Krasikov et al. (2012) investigated the global gene expression in *Synechocystis* at 0, 6, 12, 24, and 96 h during nitrogen starvation with microarrays. Their transcriptome data can be retrieved from the URL^2^. We therefore investigated the correlation between our DNA methylation data of N72 and their transcriptome data at 96 h after nitrogen starvation by comparing their differentially expressed genes (DEG) with the DMR-related genes found in our study.

## Results

### Whole-Genome Methylation Landscape in *Synechocystis*

Our sequencing data has been deposited in NCBI with Bioproject ID PRJNA445519^3^. About 1.5, 1.76, and 2.06 Gb clean data with read length longer than 125 bp were generated for NC, N72, and NR, respectively, and the sequencing depth reached 380×. The bisulfite conversion rate was 99.72, 99.70, and 99.71% for NC, N72, and NR, respectively, showing almost complete bisulfite conversion. The unique mapped reads accounted for 75.61, 81.05, and 71.79% of the clean reads in NC, N72, and NR, respectively, resulting in a median coverage of 163 reads per base.

As shown in **Table 1**, the overall methylation levels of the three samples were similar. 1.35, 1.34, and 1.48% of cytosines were methylated for NC, N72, and NR, respectively. About 3.0% CG, 0.9% CHG, and 1.0% CHH were methylated comprising the DNA methylation in each of the samples. In total, 96,961 sites for methylcytosine (thereafter mC sites) were identified in the *Synechocystis* genome, including 62,415, 18,057, and 16,489 in CHH, CG, and CHG context (H represents any nucleotide except guanine), respectively. For all the three samples CHH was always the most preferred context by mC site, followed by CG and CHG, e.g., mC sites in CHH, CG, and CHG context accounted for about 63, 21, and 16% of the total sites, respectively (**Supplementary Table S2**). Interestingly, large difference in the number of mC site was found between samples despite their similar overall methylation level (**Supplementary Table S2**). Under normal nitrogen 79,801 mC sites were found, but the number decreased to 56,914 after nitrogen starvation for 72 h. Interestingly, after 12 days of nitrogen recovery, the number of the site (58,884) was still close to that under nitrogen starvation, much less than under normal nitrogen.

**Table 1:** Proportion of 5-methylcytosines under normal nitrogen (NC), nitrogen starvation (N72), and nitrogen recovery (NR).

Samples	Proportion of	Proportion of	Proportion of	Proportion of
	methylated CG (%)	methylated CHG (%)	methylated CHH (%)	all mC (%)
NC	3.05	0.90	1.03	1.35
N72	3.02	0.90	1.03	1.34
NR	3.15	1.00	1.16	1.48


We also notice that methylation site ratio (sites for methylcytosine vs. plasmid nucleotides) differs among plasmids and chromosome. For instance, the methylation site ratio of mC site in CG, CHG, and CHH context in pSYSG_M was about 6.3, 4.4, and 16.5%, respectively, vs. only about 0.4, 0.4, and 1.3% in chromosome (**Supplementary Figure S1**). More extreme, there were no mC detected in pSYSA_M.

### Significant Transgenerational Epigenetic Inheritance in Cyanobacterium

**FIGURE 1:**
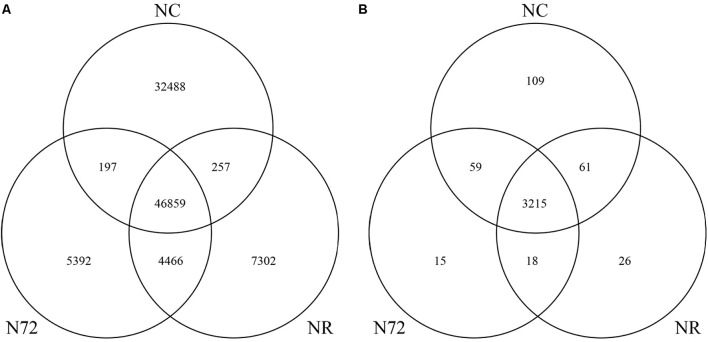
Venn diagram showing the overlapped mC sites **(A)** and the overlapped genes with methylcytosine **(B)** among normal nitrogen (NC), nitrogen starvation (N72), and nitrogen recovery (NR).

To look into the details of the similar numbers of mC site in NR and N72, Venn diagram was drawn. As shown in **Figure 1A**, after nitrogen starvation 32,745 mC sites (32,488 plus 257) which had been found in NC disappeared and 9,858 new sites (5,392 plus 4466) were induced. Relatively small change in mC site occurred after cells were transferred from nitrogen deficiency to nitrogen recovery, with 5,589 sites (5,392 plus 197) disappearing and 7,559 sites (7,302 plus 257) newly induced. There were 47,056 (46,859 plus 197) overlapped sites between NC and N72, 51,325 (46,859 plus 4,466) between N72 and NR, and 47,116 (46,859 plus 257) between NR and NC. Of these overlapped sites, 46,859 were detected in all the three samples, and these sites were possibly less susceptible to the nitrogen treats than other mC sites. Of the 9,858 sites newly induced by nitrogen starvation stress, more than 45% (4,466 sites) were preserved in NR. On the other hand, of the 32,745 mC sites which had been found in NC but disappeared in N72, NR only regain about 1% (257 sites). These indicated that the profile of mC sites in NR was much more similar with N72 than NC.

**FIGURE 2:**
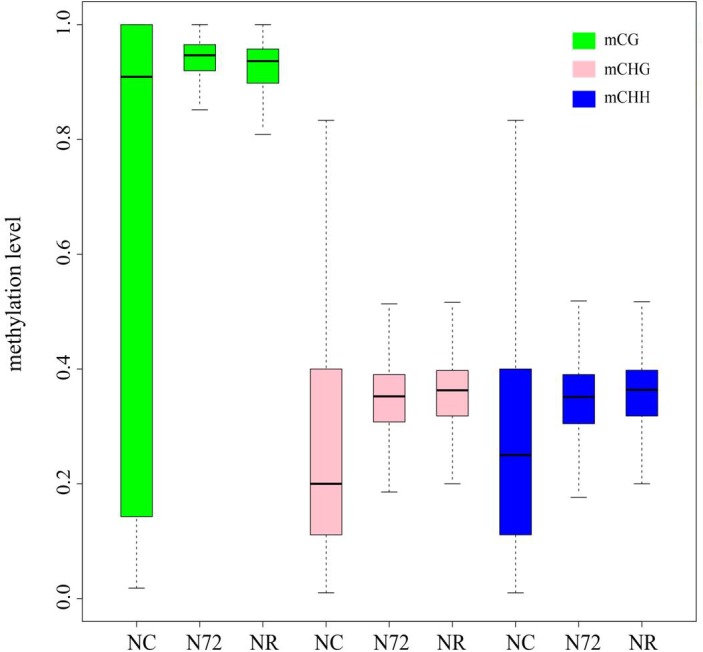
Box plot showing the methylation level of sites for methylcytosine under normal nitrogen (NC), nitrogen starvation (N72), and nitrogen recovery (NR). Distribution of methylation level in nitrogen starvation (N72) is similar with that in nitrogen recovery (NR) rather than in normal nitrogen (NC).

Considering the similar overall methylation level among the three samples (**Table 1**) and the decreased numbers of mC sites in NR and N72, mC sites in NR and N72 must have higher methylation level than those in NC. Analysis of methylation level of mC sites confirmed this, and further supported the similarity in methylation pattern between NR and N72. Compared with NC, mC site in N72 showed median-increased but variability-decreased methylation level distribution (**Figure 2**). Furthermore, similar methylation level distributions were found for mC site in N72 and that in NR (**Figure 2**). With regard to site context, we found the median of methylation level was much higher in CG context than in non-CG context (CHG and CHH context).

### Methylated Genes

All mC sites were annotated in this study, and we found a total of 3,503 genes containing mC, unexpectedly as high as 95% of all genes in *Synechocystis*. There were 3,444, 3,307, and 3,320 genes with mC in NC, N72, and NR, respectively (**Supplementary Table S3**). As shown in **Figure 1B**, after nitrogen starvation 170 genes (109 plus 61) lost mC while 33 new genes (15 plus 18) acquired mC sites. In addition, 74 genes (59 plus 15) lost and 87 genes (61 plus 26) acquired mC sites further after cells were transferred from nitrogen deficiency to nitrogen recovery.

We further investigated the methylation level of mC sites within each functional region, i.e., gene body, intergenic region (region between genes), upstream region (promoter and 5′ UTR) and downstream region (3′ UTR). As shown in **Supplementary Figure S2** no obvious difference in methylation level distribution could be found between functional regions.

### Methylation in Transposable Elements

Transposable elements (TEs) are hyper-variable in cyanobacterial genomes, and it could work as a new perspective to further explore the diversity of cyanobacteria in the ever changing environment (Lin et al., 2011; Wang et al., 2015). We investigated the methylation of mC sites exclusively within TEs (thereafter TE-type mC sites). In total, 497 TE-type mC sites were detected in 107 TEs. In NC there were 338 distributed in 95 TEs, and after nitrogen starvation the sites decreased to 150 distributed in 65 TEs. After nitrogen was recovered 130 TE-type mC sites were found within 64 TEs (**Supplementary Figure S3**).

We also investigated the methylation level of the TE-type mC site. Similarly, TE-type mC sites showed increased median methylation level except those in CG context after nitrogen starvation. Even so, TE-type mC sites have distinct nitrogen starvation-response pattern, i.e., a much wider methylation level range were found for TE-type mC sites except those in CHG context after nitrogen starvation, as opposed to what we had showed when mC sites of all types were taken into account (**Figure 2** and **Supplementary Figure S4**). Additionally, we found methylation level distribution differed between N72 and NR at TE-type mC sites in CG and CHH context, namely the median methylation levels at these two TE-type sites were much higher in NR than in N72 (**Supplementary Figure S4**).

### Differentially Methylated Region Analysis

Due to the large difference in pattern of DNA methylation between N72 and NC, the DMRs were analyzed. After nitrogen deficiency a total of 1,194 genes were found in DMRs, with 1,042 genes in hyper-methylated regions and 152 genes in hypo-methylated regions. By KEGG enrichment analysis, we found the genes in hyper-methylated regions were mainly enriched in biosynthesis of antibiotics, biosynthesis of secondary metabolites, microbial metabolism in diverse environments, and metabolic pathways. On the other hand, the genes in hypo-methylated regions were mainly enriched in porphyrin and chlorophyll metabolism, streptomycin biosynthesis and carbon metabolism (**Supplementary Figure S5**).

### DNA Methylation and Gene Expression

**FIGURE 3:**
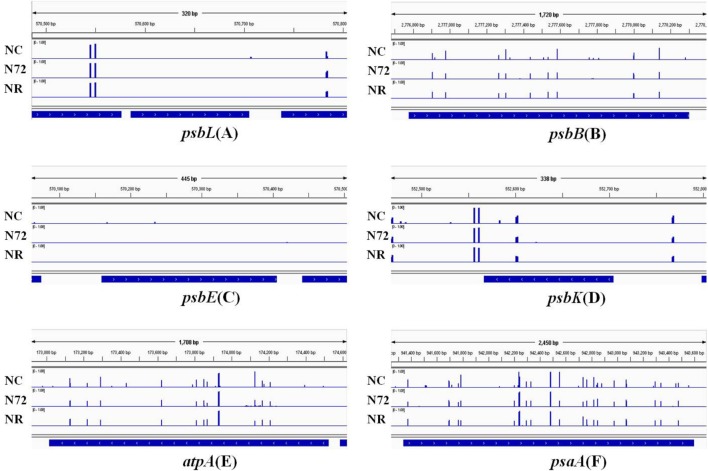
DNA methylation status of six photosynthesis related genes under normal nitrogen (NC), nitrogen starvation (N72), and nitrogen recovery (NR). Gene body and 100 bp gene-flanking regions at upstream and downstream were covered. DNA methylation status of *psbL* is shown in **(A)**, *psbB* in **(B)**, *psbE* in **(C)**, *psbK* in **(D)**, *atpA* in **(E)**, and *psaA* in **(F)**. Height of blue bars represents the methylation level of each site for methylcytosine. No obvious difference in methylation was found among the three samples.

**FIGURE 4:**
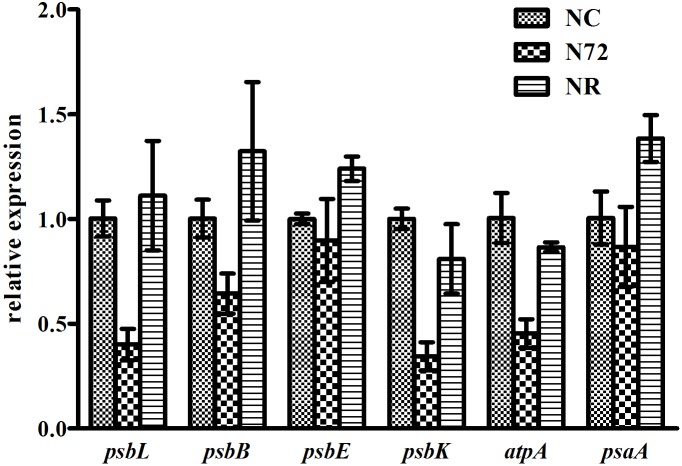
Gene expressions under normal nitrogen (NC), nitrogen starvation (N72), and nitrogen recovery (NR). *rnpB* was used as internal reference. Data represent means ± SDs from three biological replicates. Expressions of all the six genes were down-regulated under nitrogen deficiency and almost restored to normal expression level when nitrogen was recovered.

The photosynthesis system, which is sensitive to nitrogen starvation and other environmental stresses, was down-regulated by most stresses (Qiao et al., 2013). Six photosynthetic genes (*psbL*, *psbB*, *psbE*, *psbK*, *atpA*, and *psaA*) were checked for their methylation status. *psbL* and *psbE* contained no or few mC sites whereas the other four contained many hyper-methylated mC sites (**Figure 3**). For all the six genes, the three samples showed similar methylation pattern (**Figure 3**). We also measured the expression of the six genes mentioned above. As shown in **Figure 4**, for all the six genes the expression was down-regulated upon nitrogen deficiency, and almost restored to normal expression level after nitrogen was recovered, regardless of the similarity in DNA methylation among three samples.

We also investigated the correlation between our DNA methylation data of N72 and the published transcriptome data at 96 h after nitrogen starvation (Krasikov et al., 2012). Our DMRs-related genes whose expression can be identified in the data were shown in Supplementary Data Sheet S1. Of the 1,042 genes in hyper-methylated regions, expression of 987 genes was identified in their transcriptome data, including 488 genes with down-regulated expression, 493 genes with up-regulated expression, and 6 genes with no changed expression. Likewise, of the 152 genes in hypo-methylated regions, 134 genes were found to express, including 64 genes with down-regulated expression, and 70 genes with up-regulated expression.

### Growth of NC and NR Under Limited Nitrogen

To explore whether the transgenerational epigenetic modifications could benefit NR when encountering next time limited nitrogen resource, we recultured NC and NR in limited nitrogen (N^1/3^ BG11), and compared their growth. Specific growth rates of NC and NR in N^1/3^ BG11 were shown in **Supplementary Figure S6**. The specific growth rate was 0.194 ± 0.002 d^-1^ for NC, vs. 0.196 ± 0.001 d^-1^ for NR. Even though slightly higher growth rate seemed in NR, no statistically significant difference could be found (*p* > 0.05).

## Discussion

With SMRT sequencing, many methylomes were explored in bacterial. At first, Clark et al. (2012) introduced plasmids containing cloned distinct DNA methyltransferases into *E. coli* mutant lacking endogenous DNA methyltransferases, and examined the methylation status of the plasmids. Then they investigated the methylation status of six bacterial genomes with the same technology, including *Geobacter metallireducens* GS-15, *Chromohalobacter salexigens*, *Vibrio breoganii* 1C-10, *Bacillus cereus* ATCC 10987, *Campylobacter jejuni* subsp. jejuni 81-176, and *C. jejuni* NCTC 11168 (Murray et al., 2012). Finally, Blow et al. (2016) investigated DNA modifications among 230 diverse bacterial and archaeal species and found lots of reproducibly methylated target motifs. All these studies have been largely limited to m^6^A and m^4^C due to the weak and somewhat diffuse SMRT signal generated by m^5^C (Flusberg et al., 2010). The first genome-wide study of m^5^C at base resolution was carried out by applying WGBS into *E. coli* and found cytosine methylation may take part in regulation of gene expression in stationary phase as a regulator (Kahramanoglou et al., 2012). With respect to cyanobacteria, SMRT sequencing showed the existence of m^6^A in *Microcystis* (Zhao et al., 2017), and Hagemann et al. (2018) profiled the global DNA methylation (including m^6^A, m^4^C, and m^5^C) in *Synechocystis* by using combination of SMRT sequencing and WGBS, which indicated 5-methylcytosine and m^4^C in *Synechocystis* were in the context of m^5^CGATCG and GGm^4^CC, respectively. All these studies were conducted to determine the specificity of methyltransferases.

In our study, the CG methylation indeed corresponds to the m^5^CGATCG, and CHG and CHH methylations comprise the GGm^4^CC. Considering the methylation level change of CHG obviously differed from that of CHH in TEs (**Supplementary Figure S4**), we proposed the differential influence of bases downstream the methyltransferase target motif (GGm^4^CC), and therefore distinguished CHG from CHH. A similar situation for the differential influence of base outside of the target motif was found in *E. coli* K12 of mid-exponential phase, where the methylation level for CmCCWGG was lower than [ATG]mCCWGG (mCCWGG was the methyltransferase target motif) (Kahramanoglou et al., 2012). Due to the partially resistance of m^4^C to the bisulfite-mediated deamination, the methylation level of GGm^4^CC context is much lower than that of m^5^CGATCG (Hagemann et al., 2018). Consistent with this, our study also showed the methylation level of mCG methylation was higher than those of mCHG and mCHH.

Some features in *Synechocystis* were also identified in this study. Firstly, we found about 3.0% CG, 0.9% CHG, and 1.0% CHH were methylated in *Synechocystis*, which is different from chordata (31.1–80.3% CG, 0.17–1.22% CHG, and 0.12–0.91% CHH were methylated) and magnoliophyta (22.3–59.4% CG, 5.92–20.9% CHG, and 1.51–3.25% CHH were methylated) (Cokus et al., 2008; Feng et al., 2010), but similar with the eukaryotic microalga *Chlamydomonas reinhardtii* (5.4% CG, 2.6% CHG, and 2.5% CHH were methylated) (Feng et al., 2010). Then, we found no obvious differences in methylation level distribution among functional regions, which maybe implies that there is no preference of hyper/hypo-methylation to a certain functional region in *Synechocystis*. In contrast, in eukaryotic microalga *Chlorella variabilis*, CG within gene body is universally methylated while CG within promoter region is seldom methylated (Zemach et al., 2010). These differences are maybe due to the lack of nucleosomal structure and different protein factors in *Synechocystis*. It is also reported that hyper-methylated CG in nucleosome linkers is involved in nucleosome positioning and chromatin compaction (Huff and Zilberman, 2014).

In eukaryotes (Lindahl, 1981; Bird, 2002) and some bacteria such as *E. coli* (Roberts et al., 1985; Kahramanoglou et al., 2012; Militello et al., 2014) and *Helicobacter pylori* (Kumar et al., 2012, 2018) there are evidence for the regulation role of DNA methylation on gene expression. However, when the correlation between our DNA methylation data of N72 and the published transcriptome data at 96 h after nitrogen starvation (Krasikov et al., 2012) was investigated, neither preference of hyper-methylated region to down-regulated expression nor preference of hypo-methylated region to up-regulated expression were found, we therefore speculate that there was probably no correlation between methylation status and gene expression in *Synechocystis*. Also, we found the expression of some photosynthesis-related genes seem to be independent of their methylation status in *Synechocystis*.

With applying methylation sensitive amplified polymorphism analysis to *Oryza sativa* L, Kou, H.P. has proved a whole-generation nitrogen deficiency stress (10 or 20 mg L^-1^ nitrogen) could induce methylation alteration in the leaf-tissue of the stressed plants (Kou et al., 2011). In addition, with multiple electrophoresis-based polymorphism analysis techniques Yu et al. (2013) reported that nitrogen addition stress could also induced alterations in cytosine methylation patterns in natural populations of *Leymus chinensis*. However, these electrophoresis technology-based studies cannot provide the details about methylation change at base resolution. To investigate whether DNA methylation in bacterial could be influenced by surrounding environment, and if influenced, whether the altered DNA methylation pattern is inheritable, we compared the global DNA methylation among normal nitrogen (NC), nitrogen starvation (N72), and nitrogen recovery (NR) in the model cyanobacteria, *Synechocystis*. After 72 h of nitrogen deficiency about 40% mC sites were demethylated, but the methylation level of the remaining mC sites increased, which results in the almost unchanged overall methylation level. Because most *Synechocystis* cells can become dormant and survive as long as 45 days under nitrogen deficiency (lab observation), the altered DNA methylation pattern in N72 reflects the cellular response to nitrogen deficiency rather than the death or enrichment of some subpopulation. Conclusion can therefore be drawn that in cyanobacteria, environment stress could induce alternations in global DNA methylation pattern through reducing the number of mC sites but increasing the methylation level of the remaining mC sites.

The similar DNA methylation pattern in NR and N72 is interesting. Considering the cell doubling time for *Synechocystis* is about 23 h, cells in NR sample have already propagated for about 12 generations since being transferred into normal nitrogen, i.e., one cell propagates for 12 times and becomes into 4,096 cells. However, NR regained only 1% of mC sites which demethylated after nitrogen deficiency and preserved about 45% of mC sites induced by nitrogen deficiency. Furthermore, mC sites in NR preserved high levels of methylation as those in N72. All these implied that the altered DNA methylation pattern induced by nitrogen deficiency stress could be partly inheritable even though the environment stress was removed, which demonstrates the environmental memory of epigenetic modifications in cyanobacteria, as in higher plants (Henderson and Jacobsen, 2007).

Environmental stress could generate particular defense epigenetic changes across generations in plants (Holeski et al., 2012), and in *Oryza sativa* L, alteration in DNA methylation induced by nitrogen deficiency stress could contribute the resistance to the nitrogen stress (20 mg L^-1^ nitrogen) for the non-stress suffered progenies which inherited the altered methylation pattern (Kou et al., 2011). The physiological significance of mC in bacterial genomes has raised enigmatic questions during several decades (Sanchez-Romero et al., 2015). Even though slightly higher growth rate seemed in NR cells, no significant growth difference between NC and NR can be found when being recultured in nitrogen limited condition. Considering that nitrogen deficiency stress lasts only 72 h, we speculate DNA methylation alteration in NR is not enough to increase the adaptation to nutrient limitation significantly. In the future examination of growth and genome-wide DNA methylation of *Synechocystis* having suffered nitrogen deficiency repeatedly or long term of nitrogen deficiency would provide more information. Furthermore, due to WGBS used in this study could not cover N^6^-methyladenine, combination of SMRT sequencing and WGBS will therefore be needed for more comprehensive understanding of epigenetic adaptation to the ever changing environment in *Synechocystis*.

In summary, we confirmed the widely spread mC methylation and response of DNA methylation to nutrient depletion in *Synechocystis*. What’s more, we verified the modified DNA methylation pattern could be partly inheritable in cyanobacteria. The results of this study might provide a primer to explore the wealth of information on the epigenetic transgenerational inheritance in prokaryotes.

## Author Contributions

JW contributed to conception and design of the study. LH, MD, PX, ZC, and YJ performed the experiments. LH, AL, and PX performed the statistical analysis. JW and LH wrote the first draft of the manuscript. JW, LH, and PX wrote the sections of the manuscript. YJ, MD, ZC, HL, and ZH contributed to manuscript revision. All authors read and approved the submitted version.

## Conflict of Interest Statement

The authors declare that the research was conducted in the absence of any commercial or financial relationships that could be construed as a potential conflict of interest.

## Footnotes

1 https://github.com/xflicsu/swDMR

2 http://dx.doi.org/10.1111/j.1399-3054.2012.01585.x

3 https://www.ncbi.nlm.nih.gov/

## Supplementary Material

FIGURE S1 Methylation site ratio (sites for methylcytosine vs. plasmid nucleotides) of chromosomes and plasmids under normal nitrogen **(A)**, nitrogen starvation **(B)**, and nitrogen recovery **(C)**. No methylcytosine was detected in pSYSA_M, and pSYSG_M had the highest methylation site ratios in all the three samples.

FIGURE S2 Box plot showing methylation level of sites for methylcytosine within gene body, intergenic region, upstream, and downstream region under normal nitrogen (NC), nitrogen starvation (N72), and nitrogen recovery (NR). No obvious difference in distribution of methylation level among different functional regions could be found.

FIGURE S3 Venn diagram showing overlapped mC sites exclusively within transposable elements (TEs) **(A)** and overlapped TEs with mC sites **(B)** among normal nitrogen (NC), nitrogen starvation (N72), and nitrogen recovery (NR).

FIGURE S4 Box plot showing methylation level of mC sites exclusively within TEs under normal nitrogen (NC), nitrogen starvation (N72), and nitrogen recovery (NR). Sites for methylcytosine under nitrogen starvation have higher-median and wider-range methylation level than those under normal nitrogen.

FIGURE S5 The KEGG pathway enrichments of genes in hyper-methylated regions **(A)** and genes hypo-methylated regions **(B)** after nitrogen starvation.

FIGURE S6 Specific growth rates of NC sample and NR sample re-cultured in N^1/3^ BG11. Data represent means ± SDs from three biological replicates. The difference between the two samples was not significant (*p*> 0.05).

TABLE S1 Primers used in this study.

TABLE S2 Numbers and proportions of mC sites in each context under normal nitrogen (NC), nitrogen starvation (N72), and nitrogen recovery (NR).

TABLE S3 Numbers and proportions of genes with mC sites under normal nitrogen (NC), nitrogen starvation (N72), and nitrogen recovery (NR).
