# Supplementary figures and images for: Transgenerational Epigenetic Inheritance Under Environmental Stress by Genome-Wide DNA Methylation Profiling in Cyanobacterium
Source: Front Microbiol. 2018 Jul 4;9:1479. doi: 10.3389/fmicb.2018.01479 (PMC6039552; doi:10.3389/fmicb.2018.01479)

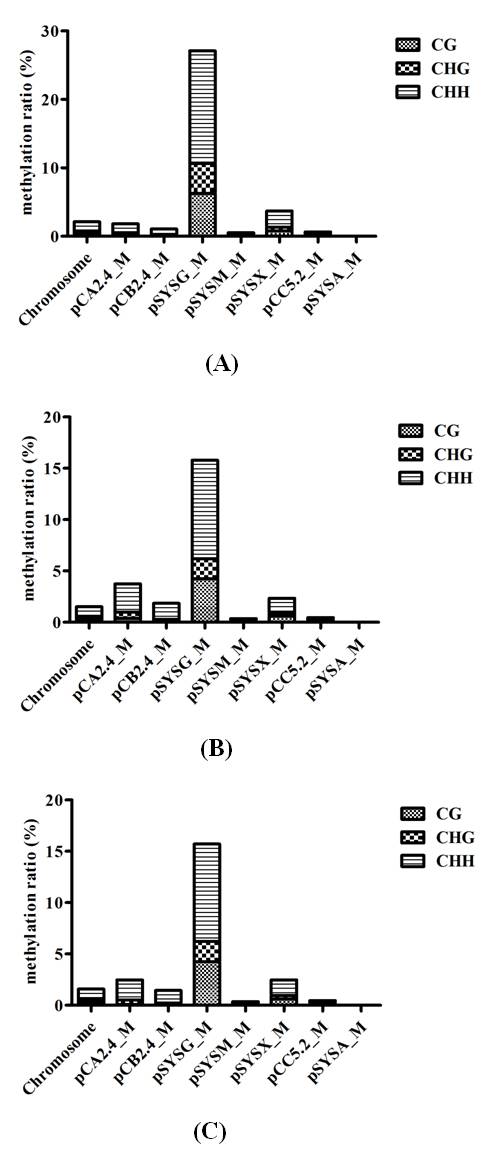

Supplement: FIGURE S1 — Methylation site ratio (sites for methylcytosine vs. plasmid nucleotides) of chromosomes and plasmids under normal nitrogen (A), nitrogen starvation (B), and nitrogen recovery (C). No methylcytosine was detected in pSYSA_M, and pSYSG_M had the highest methylation site ratios in all the three samples. [file Image_1.JPEG]

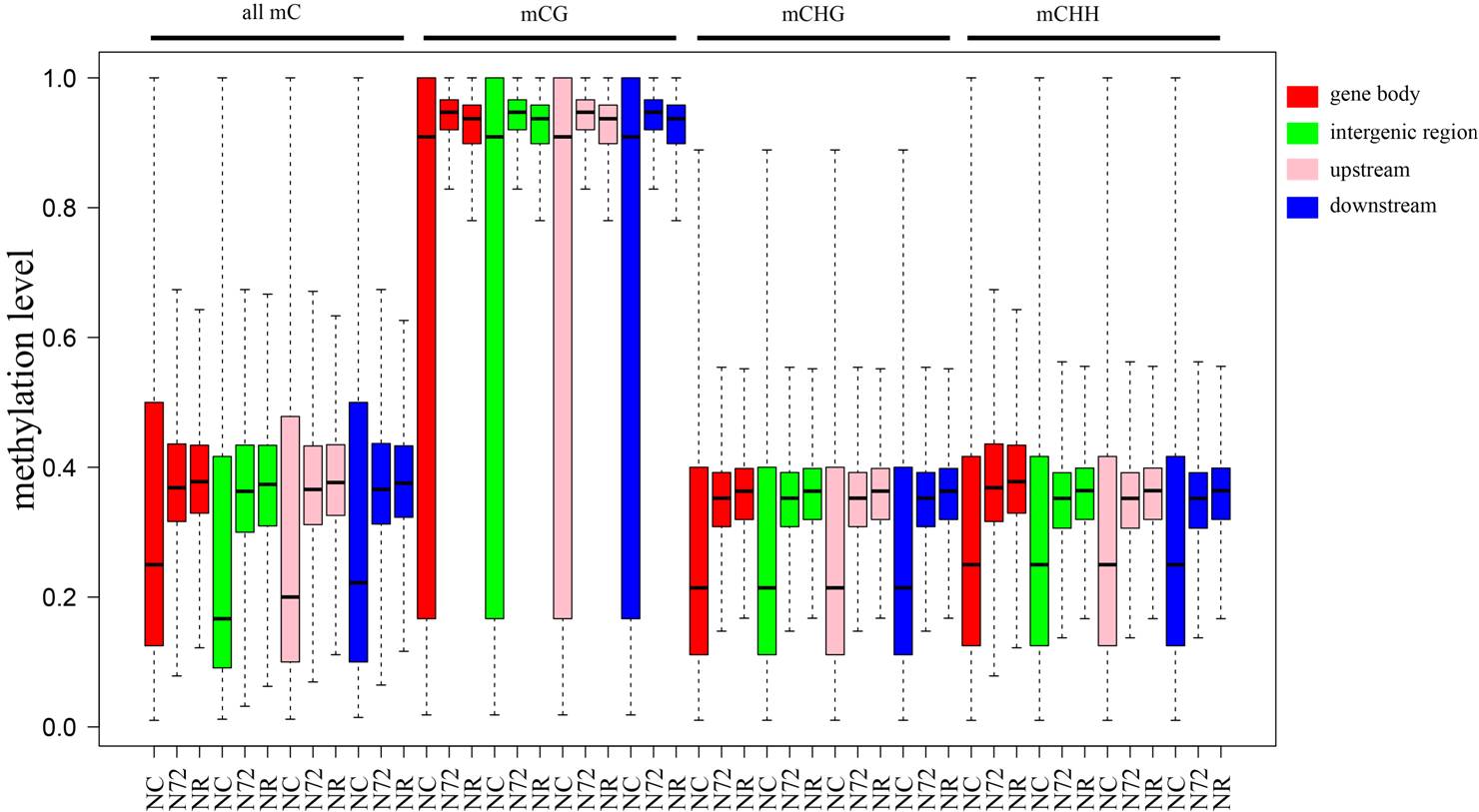

Supplement: FIGURE S2 — Box plot showing methylation level of sites for methylcytosine within gene body, intergenic region, upstream, and downstream region under normal nitrogen (NC), nitrogen starvation (N72), and nitrogen recovery (NR). No obvious difference in distribution of methylation level among different functional regions could be found. [file Image_2.JPEG]

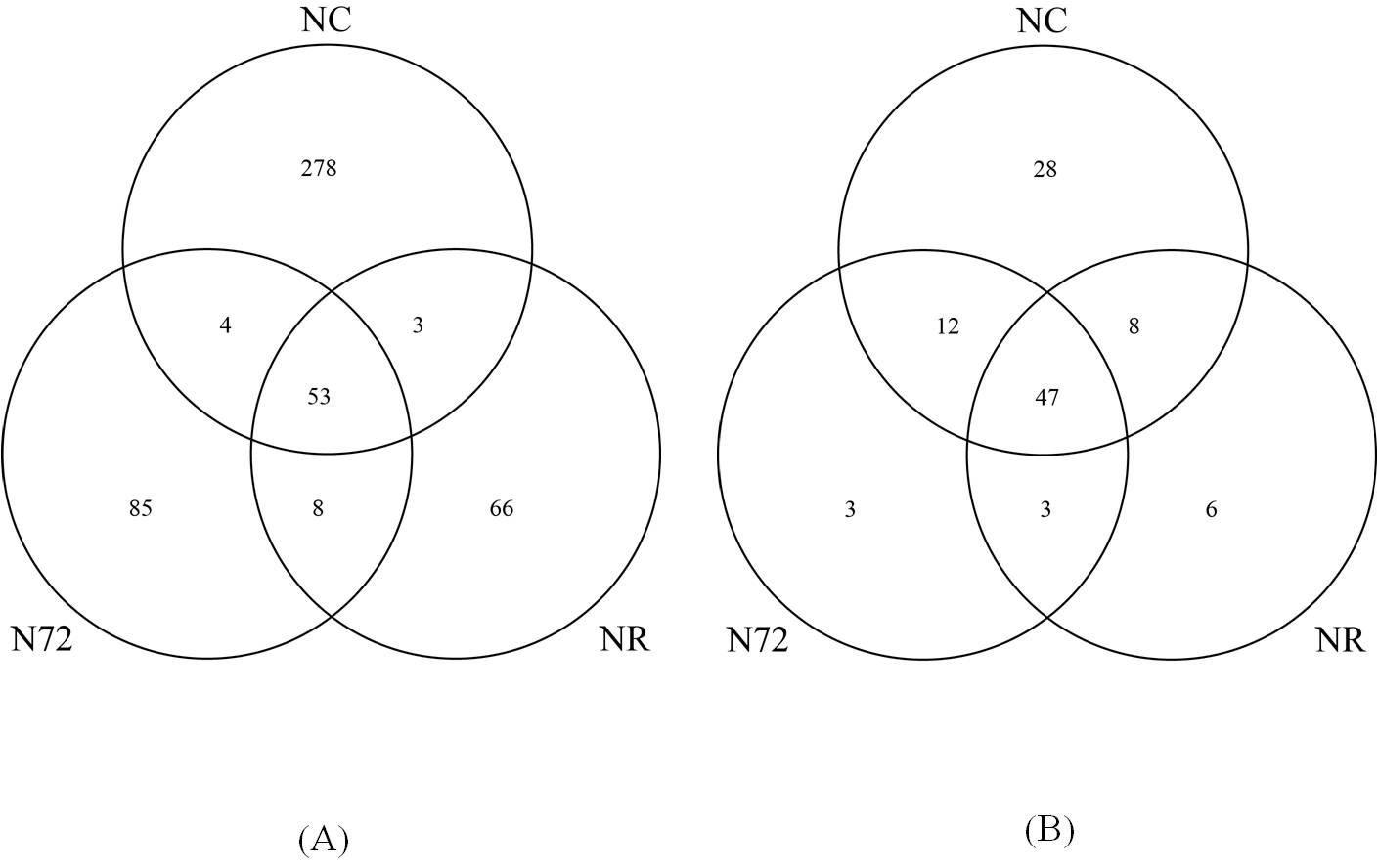

Supplement: FIGURE S3 — Venn diagram showing overlapped mC sites exclusively within transposable elements (TEs) (A) and overlapped TEs with mC sites (B) among normal nitrogen (NC), nitrogen starvation (N72), and nitrogen recovery (NR). [file Image_3.JPEG]

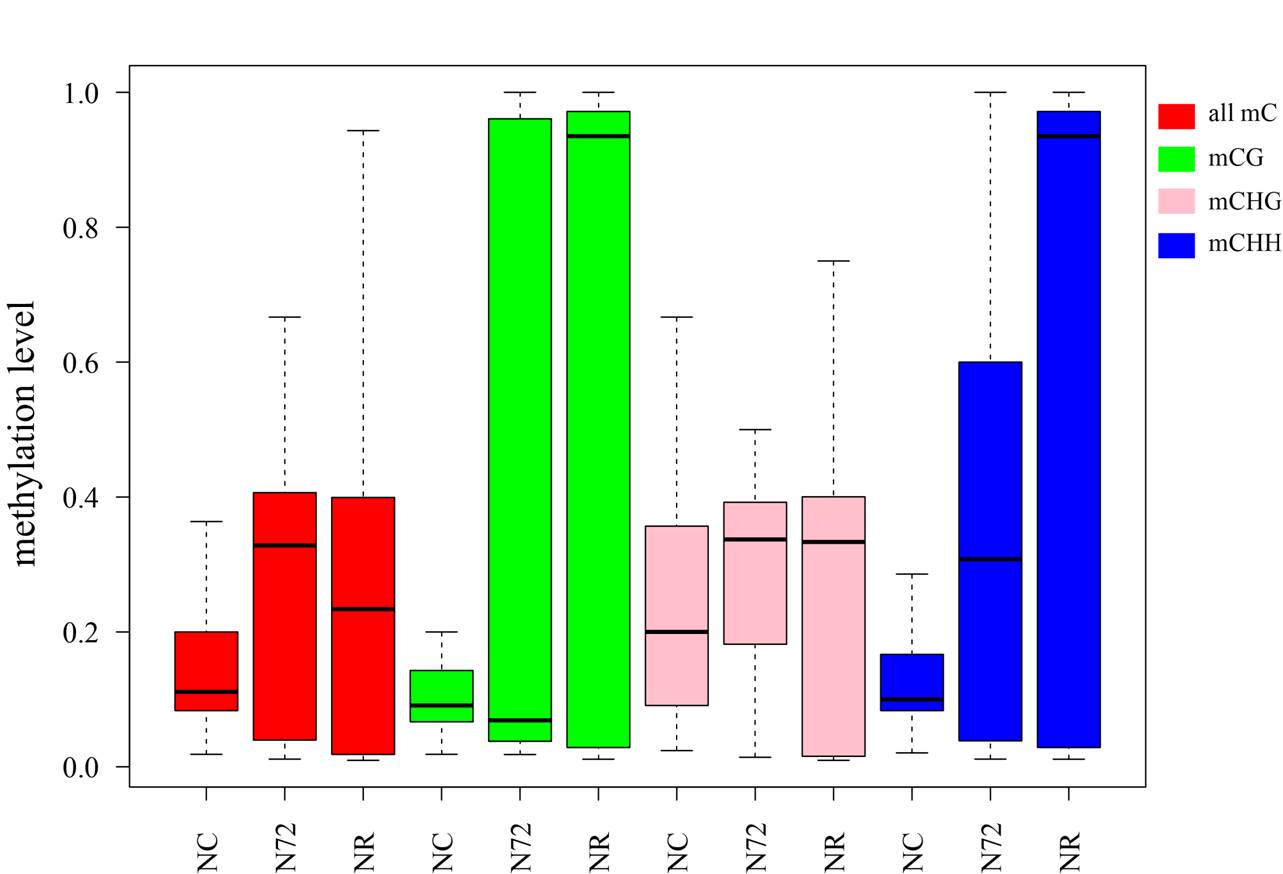

Supplement: FIGURE S4 — Box plot showing methylation level of mC sites exclusively within TEs under normal nitrogen (NC), nitrogen starvation (N72), and nitrogen recovery (NR). Sites for methylcytosine under nitrogen starvation have higher-median and wider-range methylation level than those under normal nitrogen. [file Image_4.JPEG]

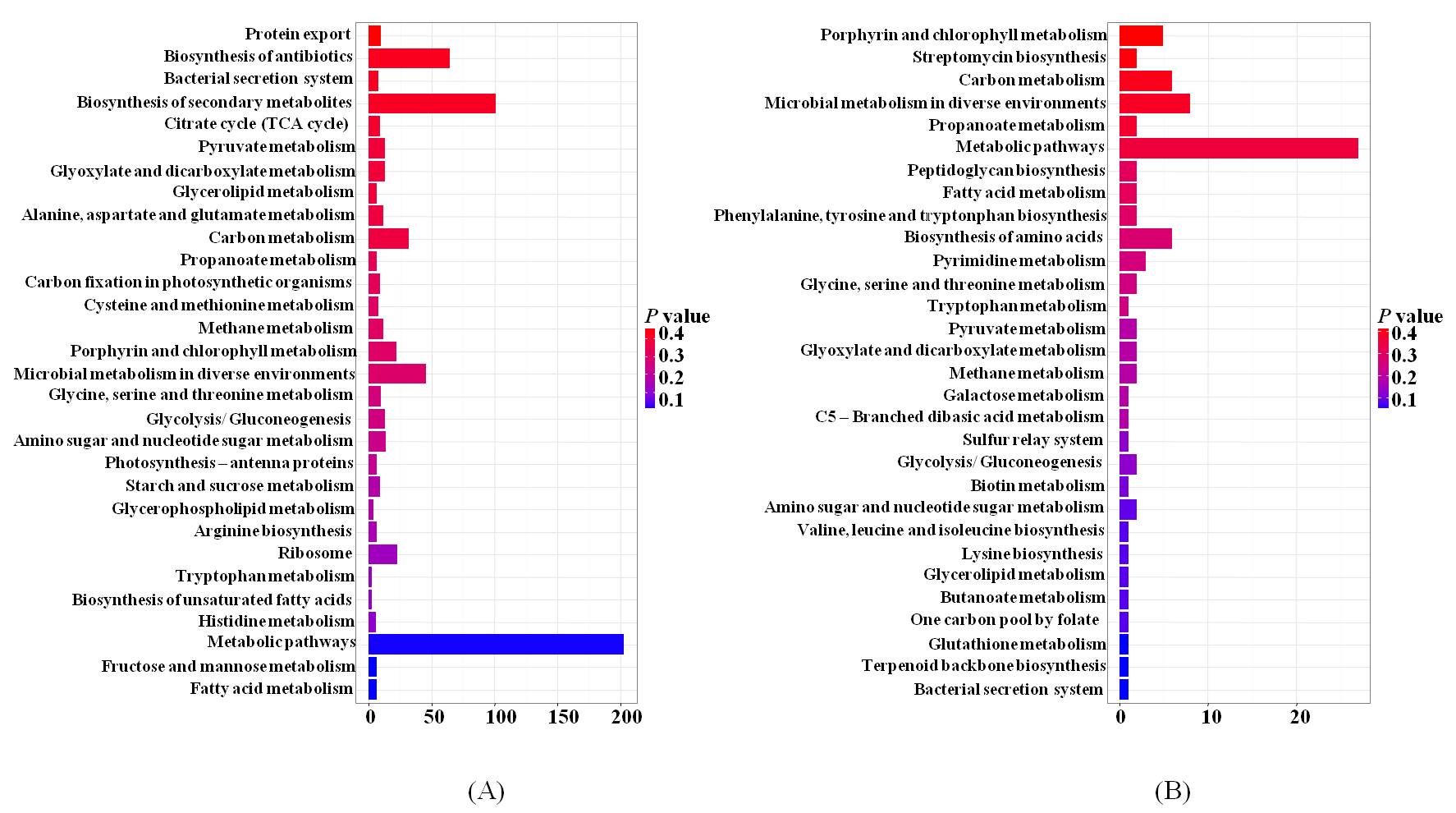

Supplement: FIGURE S5 — The KEGG pathway enrichments of genes in hyper-methylated regions (A) and genes hypo-methylated regions (B) after nitrogen starvation. [file Image_5.JPEG]

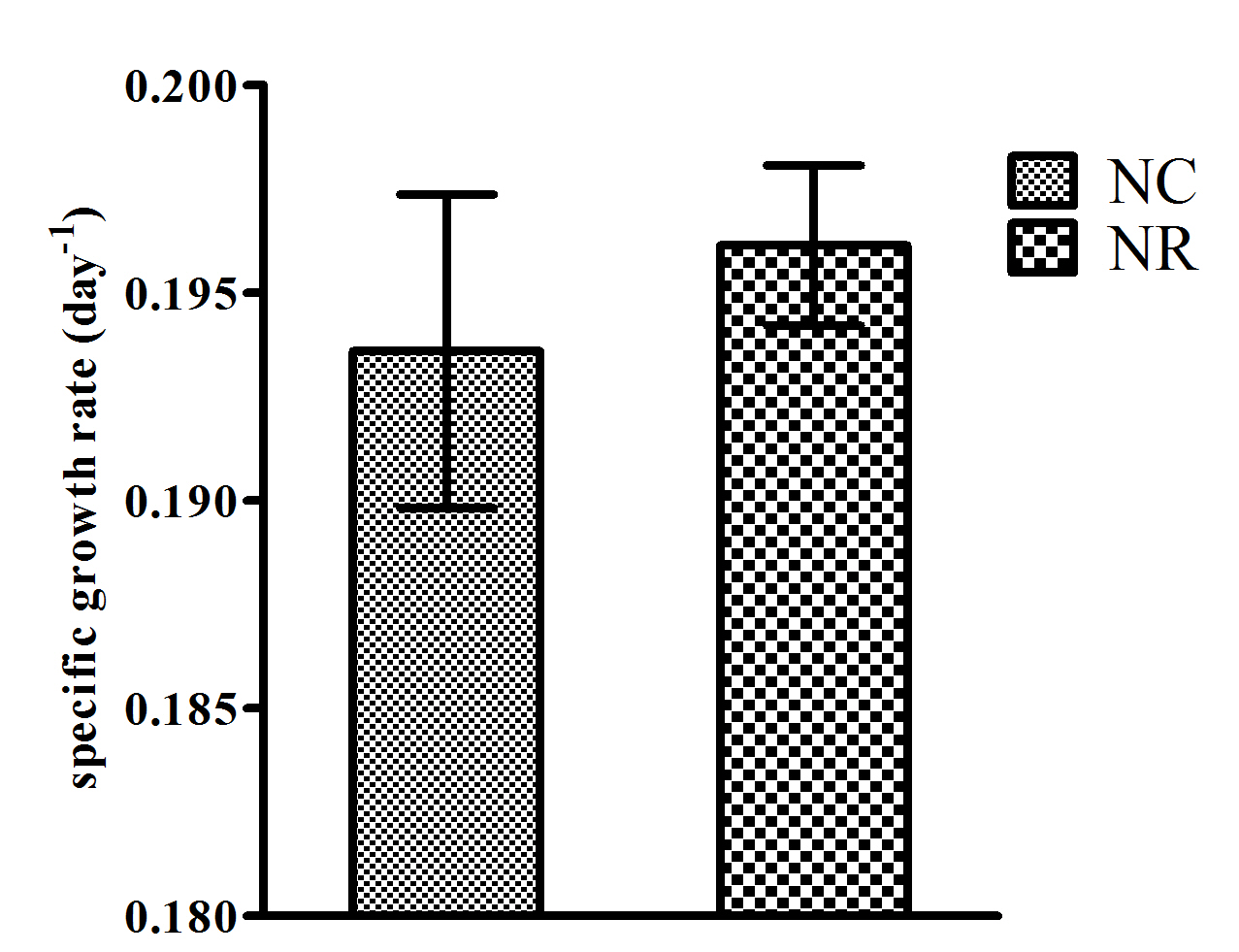

Supplement: FIGURE S6 — Specific growth rates of NC sample and NR sample re-cultured in N1/3 BG11. Data represent means ± SDs from three biological replicates. The difference between the two samples was not significant (p> 0.05). [file Image_6.JPEG]
