# Supplementary material for: Transgenerational Epigenetic Inheritance Under Environmental Stress by Genome-Wide DNA Methylation Profiling in Cyanobacterium
Source: Front Microbiol. 2018 Jul 4;9:1479. doi: 10.3389/fmicb.2018.01479 (PMC6039552; doi:10.3389/fmicb.2018.01479)
Supplement: TABLE S1 — Primers used in this study. [file Table_1.DOCX]

Table S1 Primers used in this study

| Primers | Sequence |
| --- | --- |
| *rnpB-F* | GGAGTTGCGGATTCCTGTCA |
| *rnpB-R* | ACTGTTTACTGGTTGCTGTTTTCTA |
| *psbB-F* | GGCCCCCTGAGCGCTTGTAT |
| *psbB-R* | ACCACATGGTACCGGCCACG |
| *psbE-F* | CCGGCGAGCGTCCATTTTCC |
| *psbE-R* | GGCGGGGAGTGCCAAAAGCA |
| *psbL-F* | ACCGCCAACCGGTGGAATTGA |
| *psbL-R* | GGAGAACAAAATCCCCAACACAGCC |
| *psbK-F* | GCTCGCTAAATTGCCGGAAGCC |
| *psbK-R* | CGCCGCTTGCCACACAAAGG |
| *psaA-F* | GGCTCCGGCCATTCAACCC |
| *psaA-R* | GCGAGGAAGAACGCCCAGG |
| *atpA-F* | GAGCGGGCCGCCAAATTGAG |
| *atpA –R* | GCAGGACGGAAGCCAGCGTT |
