# Supplementary material for: Transgenerational Epigenetic Inheritance Under Environmental Stress by Genome-Wide DNA Methylation Profiling in Cyanobacterium
Source: Front Microbiol. 2018 Jul 4;9:1479. doi: 10.3389/fmicb.2018.01479 (PMC6039552; doi:10.3389/fmicb.2018.01479)
Supplement: TABLE S2 — Numbers and proportions of mC sites in each context under normal nitrogen (NC), nitrogen starvation (N72), and nitrogen recovery (NR). [file Table_2.DOCX]

Table S2 Numbers and proportions of mC sites in each context under normal nitrogen (NC), nitrogen starvation (N72) and nitrogen recovery (NR).

| Samples | mC site in CG context | mC site in CHG context | mC site in CHH context | Total mC site |
| --- | --- | --- | --- | --- |
| NC | 16211 | 13493 | 50097 | 79801 |
| NC % | 20.31% | 16.91% | 62.78% | 100% |
| N72 | 11908 | 8903 | 36103 | 56914 |
| N72 % | 20.92% | 15.64% | 63.44% | 100% |
| NR | 12006 | 9351 | 37527 | 58884 |
| NR % | 20.39% | 15.88% | 63.73% | 100% |
