# Supplementary material for: Transgenerational Epigenetic Inheritance Under Environmental Stress by Genome-Wide DNA Methylation Profiling in Cyanobacterium
Source: Front Microbiol. 2018 Jul 4;9:1479. doi: 10.3389/fmicb.2018.01479 (PMC6039552; doi:10.3389/fmicb.2018.01479)
Supplement: TABLE S3 — Numbers and proportions of genes with mC sites under normal nitrogen (NC), nitrogen starvation (N72), and nitrogen recovery (NR). [file Table_3.DOCX]

Table S3 Numbers and proportions of genes with mC sites under normal nitrogen (NC), nitrogen starvation (N72) and nitrogen recovery (NR).

| Samples | Genes with mC sites | Proportions of genes with mC sites (%) |
| --- | --- | --- |
| NC | 3444 | 93.43 |
| N72 | 3307 | 89.72 |
| NR | 3320 | 90.07 |
